# Development of a Multiplex Real-Time PCR to Disambiguate *Culicoides sonorensis* within *Culicoides variipennis* Complex, the Proven Vector of Bluetongue and Epizootic Hemorrhagic Disease Viruses in North America

**DOI:** 10.3390/cimb46090566

**Published:** 2024-08-29

**Authors:** Sarah-Jo Paquette, Dominic Czekay, Jessica Manalaysay, Tara Furukawa-Stoffer, Aruna Ambagala, Stacey Vigil, Nariman Shahhosseini

**Affiliations:** 1 Centre for Vector-Borne Diseases, National Centre for Animal Diseases, Canadian Food Inspection Agency, Lethbridge, AB T1J 3Z4, Canada; sarahjo.paquette@gmail.com (S.-J.P.); dominic.czekay@gmail.com (D.C.); jessica.manalaysay@inspection.gc.ca (J.M.); tara.furukawa-stoffer@inspection.gc.ca (T.F.-S.); 2 Departments of Chemistry & Biochemistry, University of Lethbridge, Lethbridge, AB T1K 3M4, Canada; 3 National Centre for Foreign Animal Diseases, Canadian Food Inspection Agency, Winnipeg, MB R3E 3M4, Canada; aruna.ambagala@inspection.gc.ca; 4 Southeastern Cooperative Wildlife Disease Study, University of Georgia, Athens, GA 30602, USA; staceyvigil@gmail.com; 5 Department of Biological Sciences, University of Lethbridge, Lethbridge, AB T1K 3M4, Canada

**Keywords:** bluetongue, epizootic hemorrhagic disease, *Culicoides variipennis* complex, *Culicoides sonorensis*, real-time PCR

## Abstract

Species delimitation of *Culicoides* complex species can be challenging. Among species within the *Culicoides variipennis* complex, *C. sonorensis* is considered the primary vector of bluetongue virus (BTV) and epizootic hemorrhagic disease virus (EHDV) in North America. Morphological identification of *C. sonorensis* within the *C*. *variipennis* complex is laborious, time-consuming, and requires entomology expertise. Therefore, in this study we developed and validated a multiplex real-time PCR for rapid detection and differentiation of *C. sonorensis* from the two other main cryptic species (*C. variipennis* and *C. occidentalis*) within the *C. variipennis* complex. The assay targets the EF1α gene and has a built-in internal control targeting 18 S. The specificity and the sensitivity of the multiplex real-time PCR were evaluated using morphologically identified reference and field-collected specimens. The multiplex PCR was 100% specific when nucleic acid extracted from *C. variipennis*, *sonorensis*, and *occidentalis* specimens was tested. When nucleic acid extracted from pools of midges was tested, the multiplex PCR was able to detect all three *Culicoides* species with comparable sensitivity. The multiplex assay, however, failed to detect eight morphologically identified *C. sonorensis* specimens collected from Alberta in 2014. The EF1α gene sequences of these specimens formed a distinct phylogenetic cluster, amongst those from *C. variipennis*, *sonorensis*, and *occidentalis*, suggesting that they belong to a different species. We hypothesize that those specimens might be *C. albertensis*, the only other species remaining in the *C. variipennis* complex with known geographical distribution in North America. We believe that this highly sensitive and specific multiplex real-time PCR assay could be an effective tool for rapid detection and differentiation of *C. sonorensis*, the known vector of BTV and EHDV, in trap collections in future vector surveillance programs.

## 1. Introduction

*Culicoides* Latreille (Diptera: Ceratopogonidae) biting midges are known to transmit arboviruses of veterinary importance, including bluetongue, epizootic hemorrhagic disease (EHD), African horse sickness, equine encephalitis, Schmallenberg, and Akabane viruses. Among these, bluetongue virus (BTV) and epizootic hemorrhagic disease virus (EHDV) are of particular interest in North America as they have been associated with significant mortality of both wild and domestic ruminant populations [1,2,3].

Bluetongue is a hemorrhagic disease mainly affecting ruminants such as sheep and cattle; however, the infection may occur in any domestic/wild ruminant. In contrast, EHD primarily causes fatal hemorrhagic disease primarily in wild ruminants [4]. The geographical range of BTV and EHDV vectors appears to be expanding in North America [5]. Recent outbreaks of BTV and EHDV, as well as the increasing number of their serotypes in North America, highlight the changing epidemiology of these diseases. Climate change has clearly altered the global distribution, vector competency, and composition of *Culicoides*, potentially promoting the expansion of exotic viruses to previously disease-free areas [6].

Canada is generally considered free of most viruses transmitted by *Culicoides* except for BTV and EHDV. The Okanagan Valley of British Columbia (BC), Canada, has experienced sporadic incursions of two serotypes of BTV; however, the virus was not documented as having been established beyond a single vector season. In October 2015, three BTV-17 seropositive Canadian beef cattle were identified in Southern Ontario (ON) [7]. Since then, Canada no longer declares itself free of BTV, but rather considers itself “seasonally free” if no positive cases are identified within a season. There is a rapid northward expansion of EHDV observed in the USA, with severe outbreaks having already been reported in states bordering Canada. In 2013, at least 50 white-tailed deer and pronghorn antelope died from a suspected EHDV outbreak in southern Alberta (AB) [8]. In September 2017, EHDV-2 was confirmed in two dead white-tailed deer found in Southern Ontario [9].

Among *Culicoides* species, *Culicoides sonorensis* (Wirth and Jones 1957) is the most well-known vector able to transmit both BTV and EHDV in North America [1]. The identification of *C. sonorensis* is challenging, as this species is within the *C. variipennis* complex belonging to the subgenus *Monoculicoides* Khalaf, a complex of closely related species that are not distinguishable from each other morphologically and genetically [10]. When originally described, *C. variipennis* complex consisted of six species, though presently, only three distinct species are recognized: *C. occidentalis* (Wirth and Jones 1957), *C. sonorensis*, and *C. variipennis* (Coquillett) 1901. The former distinct species *C. albertensis* (Wirth and Jones 1957) and *C. australis* (Wirth and Jones 1957) are now designated as a subspecies of *C. sonorensis*. Despite the current taxonomic arrangement, species identification remains difficult due to very subtle morphological differences between these complex species [11]. The inability to morphologically distinguish between synonymous species of *C. sonorensis* could contribute to the purported range extension of *C. sonorensis* [12].

DNA barcoding has been proven to be an effective strategy for the molecular identification of many taxa [13,14]. However, some studies have found that the commonly used mitochondrial cytochrome c oxidase I (COI) gene fails to differentiate closely related species of *Culicoides* biting midges within the *C. variipennis* complex [15]. This highlights the need for developing additional tools for the molecular discrimination of cryptic species within the *C. variipennis* complex. Several studies have examined the efficiency of alternative gene markers for barcoding eukaryotes, such as internal transcribed spacer (ITS), which is split into ITS1 and ITS2 [16], and the translation elongation factor 1-α (EF1α) gene. The synonymous substitutions in the EF1α gene cause a high rate of evolution [17], making it a reliable gene marker for barcoding and studying phylogenetic relationships among genera and species of eukaryotes [18].

The necessary steps for post-PCR processing (e.g., gel electrophoresis) and manual test sample scoring, which might be error-prone due to comparable product sizes generated by certain species, rendering conventional PCR techniques laborious and less efficient for large-scale surveillance projects. In contrast, TaqMan^®^ probe-based real-time PCR assays are rapid and highly sensitive and have specific molecular assays ideal for high-throughput surveillance projects with large sample sizes [19,20].

*Culicoides* biting midges are one of the least investigated and taxonomically defined groups among flies of veterinary significance worldwide. Information on species composition, distribution, and seasonality of *Culicoides* vectors is essential for understanding the epidemiology of *Culicoides*-borne diseases, for risk assessment of possible outbreaks with viruses such as BTV and EHDV, and for policy development. To accomplish this goal, the main objective of the current study was to develop and evaluate a multiplex real-time PCR assay that can detect and differentiate the three most common species within the *C. variipennis* complex. *Culicoides* midges were collected as part of a nationwide Canadian Food Inspection Agency (CFIA) surveillance program between 2014 and 2023.

## 2. Materials and Methods

### 2.1. Reference and Field-Collected Specimens

To design primers and probes for the multiplex real-time PCR, we used reference EF1α sequences obtained from morphologically characterized specimens belonging to the three most common species within the *C. variipennis* complex. The reference *C. sonorensis* specimens from the AK colony (established in 1973 using midges collected in Owyhee County, Idaho) were provided by the United States Department of Agriculture/Agricultural Research Service, Arthropod-Borne Animal Disease Research Unit. The morphologically verified (by Stacey Vigil at the Southeastern Cooperative Wildlife Disease Study, University of Georgia) reference *C. variipennis* specimens were obtained from field collections in Frontenac, Quebec, Canada. The reference *C. occidentalis* specimens, collected from Bolsa, California, USA, were kindly provided by Dr. Bradley Mullens at the University of California, Davis.

The above reference specimens were also used to optimize and validate the multiplex real-time PCR assay. To further evaluate the multiplex PCR, we also tested CFIA archived field-collected midges preserved in 70% ethanol or stored in a −70 °C freezer. These samples were collected between 2021 and 2023 in Canada. DNA was extracted from frozen specimens, and most of the archived specimens were used using a destructive method, whereas a non-destructive method was used for high-value archived specimens.

For archived specimens in ethanol, we included 21 specimens collected in the Okanagan Valley, BC (11 from 2014 and 10 from 2015), eight from Lethbridge, AB in 2014, and 11 from Winnipeg, MB (one from 2014 and 10 from 2016). As frozen specimens at −70 °C, we included 20 randomly selected specimens from McKinley, BC, in 2023. All specimens were identified and confirmed as belonging to the *C. variipennis* complex at the CFIA Lethbridge Laboratory using a morphological key. Female specimens from the subgenus *Monoculicoides* were further morphologically identified to the species level using published species descriptions. All three species within the subgenus *Monoculicoides* (Khalaf, 1954) *C. varipennis*, *C. sonorensis*, and *C. occidentalis* displayed wings with well-defined pattern of pigmentation, including a distinctive dark funnel shape on the CuA2 vein. The species are characterized by a single, C-shaped spermatheca. *C. variipennis* could be further distinguished by the third palpal segment being narrower with a small sensory pit in comparison to *C. occidentalis* and *C. sonorensis*, having a broader third palpal segment with a deep sensory pit [21,22].

### 2.2. Destructive or Non-Destructive DNA Extraction

For destructive extractions, individual *Culicoides* biting midges were placed in sterile reaction tubes, and then five Zirconia/Silica beads (2.3 mm) and 300 μL phosphate-buffered saline (PBS) buffer were added to each tube. Homogenization was performed using a TissueLyser (Qiagen, Hilden, Germany) at 50 oscillations per second for 5 min [23]. Using a DNeasy Blood and Tissue Kit (Qiagen, Hilden, Germany), the supernatant—obtained by centrifuging homogenates—was used to extract DNA. For non-destructive extraction, each specimen was incubated overnight at room temperature in 275 μL lysis buffer (containing 200 μL nuclei lysis solution, 50 μL EDTA, 20 μL Proteinase K, and 5 μL RNAse A solution) provided in the Wizard SV Genomic DNA Purification System (Promega, Madison, WI, USA). Non-destructive DNA extraction was conducted to preserve the specimens for subsequent morphological characterization and confirmation [24].

### 2.3. Development of Multiplex Real-Time PCR Assay Using Species-Specific Primers and Probes

Primers previously designed [7] were used to amplify a 1264-bp region of the EF1α gene in morphologically identified *C. sonorensis*, *C. variipennis*, and *C. occidentalis* samples. The sequence information obtained from these samples was then used to design primers and species-specific probes to differentiate the three species.

**Figure 1:**
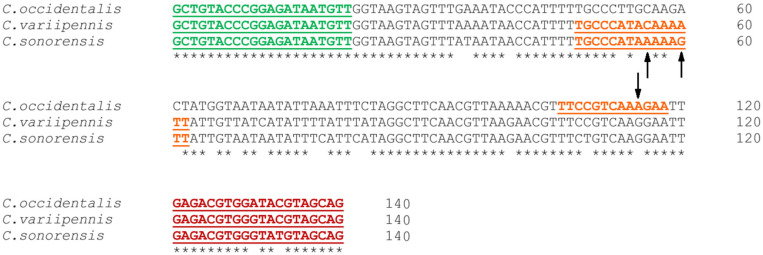
Forward and reverse primers are shown in green and red, respectively. Probe target sites are shown in orange with black arrows identifying unique single nucleotide polymorphisms for *C. sonorensis* (PP529686), *C. variipennis* (PP529717), or *C. occidentalis* (PP529684). Consensus nucleotides in the sequence alignment are indicated by an asterisk.

The extracted DNA was analyzed using a newly designed multiplex real-time PCR with the primers QMonoF (5′-GCTGTACCCGGAGATAATGTT-3′; nucleotide [nt] position 1036 to 1056 according to the *C. sonorensis* reference genome scaffold 656, GenBank accession number LN484006) and QMonoR (5′-CTGCTACRTAYCCACGTCTC-3′; nt position 1156 to 1175) to amplify 140 bp of the EF1α gene, and probes specific to either *C. occidentalis* (5′-/56-FAM/TT + C + C + GTCA + A + A + GA/3IABkFQ/-3′), *C. sonorensis* (5′-/5Cy5/TGCC + C + ATA + AAA + A + G + TT/3IAbRQSp/-3′) or *C. variipennis* (5′-/5HEX/TGCC + C + A + TA + C + AA + AATT/3IABkFQ/-3′). Each probe was labeled with a different fluorophore to differentiate all three target species in a single multiplex real-time PCR assay. Each “+” indicates the presence of an adjacent downstream locked nucleic acid, which was incorporated to increase the thermal stability and specificity of the probes. Sequence alignments of the loci used for primer design are shown in Figure 1.

For the internal control, 18S ribosomal DNA was detected using the primers 18SMonoF (5′-GGCTCAGTATATCGGCTGTAAT-3′; nt positions 2074 to 2095 according to the *C. sonorensis* reference genome scaffold 758, GenBank accession number LN484108.1), and 18SMonoR (5′-AATAATTGCACACGTTCCTT-3′; nt positions 1977 to 1996) with an expected band size of 119 bp, and the probe 18S_qProbe (5′-/56-TAMRA/AT + CA + TATA + CT + CAG + TTA + CTT/3BHQ_2/-3′) [25,26].

Multiplex real-time PCR (sono+varii+occi+18S multiplex reactions) was performed using the TaqMan Gene Expression Master Mix Kit (Thermo Fisher Scientific, Vilnius, Lithuania). The total reaction volume was 25 μL, including 12.5 µL of PCR Reaction Buffer (1× final concentration), 2.25 µL of a 5 µM QMono Primer Mix (450 nM final concentration), 0.75 µL of a 5 µM 18S Primer Mix (150 nM final concentration), 2.0 µL of a 2.5 µM TaqMan Probe Mix (200 nM final concentration of all four probes), and between 1 and 5 µL of DNA template. The final volume was made to 25 µL with the addition of nuclease-free water (IDT). The temperature profile consisted of an initial UNG incubation at 50 °C for 2 min, then DNA Polymerase Activation/UNG Inactivation at 95 °C for 10 min, followed by 40 cycles of denaturation at 95 °C for 15 s, with annealing and extension at 60 °C for 1 min. All thermal cycling was performed on a QuantStudio 7 Pro instrument (Applied Biosystems, Kingston, ON, Canada).

### 2.4. Validation of Multiplex Real-Time PCR Using Field-Collected and Reference Specimens

To confirm the ability of the newly designed multiplex real-time PCR to differentiate between *C. occidentalis*, *C. sonorensis*, and *C. variipennis*, representatives of each species were selected for testing. This included three *C. occidentalis* samples that were collected near Okanagan Falls, BC, in 2014, four *C. variipennis* samples that were collected in Winnipeg, MB, in 2016, and four *C. sonorensis* samples that were obtained from a known *C. sonorensis*-only *Culicoides* colony. Each sample was confirmed to be a member of the species it represented through barcoding of the 140 bp region of the EF1α gene (amplified by QMono primers designed in the current study), morphological identification by experts in the field, or a combination of both. Genomic DNA from each sample was obtained using bead homogenization followed by spin-column purification, and then the samples were independently tested using the multiplex real-time PCR approach.

### 2.5. Specificity and Sensitivity of Multiplex Real-Time PCR

In addition to differentiating between the three species: *C. occidentalis*, *C. sonorensis*, and *C. variipennis*, it was critical to ensure that the multiplex real-time PCR approach would not react with species of *Culicoides* outside of the *C. variipennis* complex, particularly those species that co-locate with members of the *C. variipennis* complex. To confirm this, genomic DNA extracts representing five unique species of *Culicoides* outside the *C. variipennis* complex were selected, including *C. biguttatus*, *C. obsoletus*, *C. sanguisuga*, *C. stellifer*, *C. travisi*. In order to be a useful tool for field surveillance, it is equally important for the qPCR to also be sensitive enough to detect even a single species of the *C. variipennis* complex within a larger pool of collected specimens. To test this, a single reference *Culicoid* was spiked into genomic extract pools composed of *Culicoides* outside the *C. variipennis* complex at ratios of 1:10, 1:20, and 1:30.

### 2.6. Sequencing and GenBank Submission

To obtain the EF1α sequence information, a subset of samples was subjected to conventional PCR using 18SMonoF and 18SMonoR primers. The master mix was prepared using 20.00 μL of 2X HotStarTaq Plus Master Mix (Qiagen, Hilden, Germany) and 3.20 µL of each 10 µM QMono primer (800 nM Final Concentration), and 4.00 µL of DNA template. The final volume reached 40 µL by adding nuclease-free water (IDT). The temperature profile consisted of an initial denaturation/activation at 95 °C for 5 min, followed by 40 cycles of denaturation at 94 °C for 30 s, annealing at 56 °C for 30 s, extension at 72 °C for 15 s, and a final extension at 72 °C for 1 min. The PCR products were visualized using the QIAxcel Advanced system (QIAGEN, Hilden, Germany). After cleaning the products using Zymo DNA Clean & Concentrator according to the manufacturer’s instructions, samples were sent for Sanger sequencing in both forward and reverse directions (Eurofins Scientific, Louisville, KY, USA). All sequences obtained in this study were trimmed manually, and consensus sequences were generated, which can be found under the GenBank accession numbers PP529667–PP529721.

### 2.7. Phylogenetic Tree Construction

After morphological and molecular identification using multiplex real-time PCR, a subset of specimens (*n* = 55) was included in species delineation using phylogenetic tree analysis, including 13 field-collected *C. variipennis* from Canada, 16 field-collected *C. sonorensis* from Canada, 4 *C. sonorensis* colony specimens from the USA, 17 field-collected *C. occidentalis* from Canada, and 1 field-collected *C. occidentalis* from California, USA. In the evolutionary tree study, we included four samples initially identified as *C. sonorensis/C. occidentalis* using morphological characteristics but not confirmed using multiplex real-time PCR. In addition, seven sequences were retrieved from GenBank, including three *C. variipennis* sequences from ON-2013 (KP310103, KP310106, and KP310107), one reference sequence of *C. sonorensis* generated from the KC cell line derived from the AK colony (LN484006), and three sequences from ON-2013 claimed to be *C. sonorensis* (KP310100–KP310102). The 140 bp region of the EF1α gene for all 62 sequences was aligned using Clustal Omega. A neighbor-joining (NJ) tree based on Tamura-Nei genetic distances was generated to provide a phylogeny-based delineation of different species using Geneious Prime Version 2021.2.2 [27].

## 3. Results

### 3.1. Differentiation of Species within the C. variipennis Complex Using Multiplex Real-Time PCR

Our results confirm that in a real-time multiplex qPCR experiment, each of the target species reacted specifically to the species-specific probe designed for that species (Table 1). The fluorescence of the CY5 reporter (*C. sonorensis* probe), the fluorescence of the HEX reporter (*C. variipennis* probe), and the fluorescence of the FAM reporter (*C. occidentalis* probe) increased above the threshold only when used with a reference *C. sonorensis* sample, *C. variipennis* sample, and *C. occidentalis* sample, respectively. As an internal control, the quality of all DNA extracts examined was confirmed by the amplification of 18S rDNA, detected as an exponential increase in TAMRA fluorescence above the threshold. Our results show that in-house designed probes in the multiplex assay are capable of distinguishing *C. sonorensis*, *C. variipennis*, and *C. occidentalis* from one another.

### 3.2. Specificity of Multiplex Real-Time PCR

The multiplex real-time PCR assay was effective in identifying members of the *C. variipennis* complex apart from other species of *Culicoides* with which they are known to co-locate geographically. DNA extracts of our reference *C. variipennis* complex specimens were specifically reactive to the multiplex real-time PCR, whereas extracts of *C. sanguisuga*, *C. obsoletus*, *C. biguttatus*, *C. travisi*, *C. stellifer*, *C. cockerellii*, and *C. scanloni* (all identified at the species level using COI barcoding), and extracts of *C. crepuscularis*, *C. yukonensis*, *C. downesi*, *C. riethi/gigas*, *C. wisconsinensis*, *C. denticulatus*, *C. paraimpunctatus/canadensis*, and *C. haematopotus* (all identified at the species level using a morphological key) were not (Table 2). A sigmoidal increase above the background fluorescence threshold of the *C. sonorensis* probe (CY5), *C. variipennis* probe (HEX), and *C. occidentalis* probe (FAM) identified *C. sonorensis*, *C. variipennis*, *C. occidentalis* specimens, respectively. As anticipated, no species outside of those within the *C. variipennis* complex reacted with the species-specific probes but were able to react with the 18S internal control probe, indicating a high degree of specificity (Table 2). The specificity of the multiplex real-time PCR for our target *C. variipennis*-complex species is two-fold, as an additional layer of specificity is found to be achieved by the QMono primers, which generate the amplicon bound by our species-specific probes; no species of *Culicoides* outside of the target species generated any significant amount of QMono amplicon (Supplementary Figure S1).

### 3.3. Sensitivity of Multiplex Real-Time PCR

#### 3.3.1. Sensitivity Analysis in Pools of Species outside the *C. variipennis* Complex

To be an effective tool for the surveillance of a particular *Culicoides* species, a real-time PCR assay must be capable of accurately detecting the presence of that species within a pool of field-collected samples because it is both costly and time-consuming to individually assay every collected specimen. By spiking target DNA extracts into pools of non-target DNA, we show that the multiplex real-time PCR assay described here is effective at accurately identifying the presence of DNA from the species of interest (either *C. occidentalis*, *C. sonorensis*, or *C. variipennis)*. Each target species was identified within pools of non-*C. variipennis* complex DNA containing 10, 20, and 30 samples. For pool spike-ins, Ct values for *C. sonorensis* spike-ins were the lowest on average, ranging from 28.63 to 29.89, whereas Ct values for *C. variipennis* and *C. occidentalis* spike-ins ranged from 28.97–30.31 and 34.95–36.07, respectively (Table 3). It is worth noting that all three target species were detected with similar sensitivity when comparing each pool to its respective dilution (e.g., 9 *Culicoides* plus 1 *C. occidentalis* vs. *C. occidentalis* 1/10 dilution). This demonstrates that the presence of non-target DNA did not visibly influence the sensitivity or specificity of the assay (Table 3). Furthermore, the results also demonstrate that the multiplex real-time PCR assay is highly specific for only the target species, as pools of *Culicoides* DNA extract, even including up to 29 different specimens, did not yield Ct values with any of the species-specific probes (Table 3).

#### 3.3.2. Sensitivity Analysis in Pools of Species within the *C. variipennis* Complex

It is equally important to ensure that the multiplex real-time PCR for identifying and distinguishing the target species maintains its sensitivity and specificity in cases where field collections of samples are primarily composed of the target *Culicoides* specimens. DNA from one target species (either one, two, or three specimens) was spiked into pools of DNA containing equi-volume amounts of DNA extract of a different target species; however, one that is also targeted by one of the other species-specific probes (e.g., DNA from three specimens of *C. sonorensis* spiked into a pool of DNA from seven specimens of *C. occidentalis*). Results show that regardless of which target species composes most of a sample pool, trace amounts of DNA of all targeted species remained detectable by the real-time PCR assay. Ct values of samples are observed to range from 26.87–29.90 for *C. sonorensis* spike-in, 26.14–34.53 for *C. variipennis* spike-in, and 28.83–29.86 for *C. occidentalis* spike-in. Even in situations where the DNA of one target species outnumbers the other by a factor of 9:1, both targets are detectable in every combination (Table 4). Similar to the sensitivity results in the previous pooling assay, the effect of sample pooling does not measurably change the Ct value of the spiked-in target sample(s), indicating importantly that the presence of other target species in a tested sample pool does not reduce the sensitivity or specificity of our assay. Even in situations where all three target species were present, the multiplex real-time PCR assay was successful in identifying the presence of each target (Table 4).

### 3.4. Further Validation of Multiplex Real-Time PCR Using Field-Collected Specimens of C. variipennis Complex in Canada

A subset of field specimens (*n* = 60) collected as part of a nationwide *Culicoides* biting midge surveillance program in Canada from 2014 to 2023 was subjected to the newly developed multiplex real-time PCR in order to identify them at the species level. In addition, a reference specimen of *C. sonorensis* (PP529686), *C. variipennis* (PP529717), and *C. occidentalis* (PP529684) were included as controls for the detection of each target. Apart from the eight specimens collected in Alberta, the real-time PCR assay identified all samples as belonging to one of the three targeted species (Table 5). In general, it was found that archived specimens preserved in ethanol since 2014–2016 had a higher Ct value (average Ct value 32.6) than fresh sample collections from 2023 and stored in a −70 °C freezer until processing (average Ct value 29.33) (Table 5). A very interesting shift was observed in samples collected in British Columbia, which, in the years 2014 and 2015, were found to be primarily *C. occidentalis;* however, these shifted to a majority population of *C. sonorensis* in the 2023 collection.

### 3.5. Species Delineation by Phylogenetic Tree

A phylogenetic tree was constructed using the NJ method based on 140 bp of EF1α genes from 62 sequences of the *C. variipennis* complex. The data set included 55 sequences generated from the specimens collected in different geographical regions in North America in different time periods during this study. This includes 16 *C. variipennis* sequences (8 from MB, 7 from ON, and 1 from QC), 24 *C. sonorensis* sequences (16 from BC, 3 from ON, 4 from the USA-AK colony, and 1 reference sequence derived from the AK colony), and 18 *C. occidentalis* sequences (17 from BC, and 1 sequence from the USA-California). In addition, we included four unknown sequences obtained from the *C. variipennis* complex collected in AB in 2014. Moreover, seven sequences were retrieved from GenBank.

The phylogenetic tree delineation clearly showed four distinct clusters, including three known species, *C. variipennis*, *C. sonorensis*, and *C. occidentalis*, and one outgroup. Each species of *C. variipennis*, *C. sonorensis*, and *C. occidentalis* clustered together regardless of collection site or year. Interestingly, four specimens collected from AB in 2014 during this study and three other sequences retrieved from GenBank (ON specimens collected in 2013: KP310100-KP310102) formed a distinct outgroup from the other three well-defined species within the *C. variipennis* complex. The four specimens from AB-2014 were preliminarily identified as *C. sonorensis/C. occidentalis/C. albertensis* based on morphological characteristics; however, further confirmation using the multiplex real-time PCR method confirmed their species is distinct from *C. sonorensis*, *C. variipennis*, and *C. occidentalis* (Table 5). We determined that the four specimens from AB are still part of the *C. variipennis* complex because they produced products in conventional PCR using the QMono primers after verification of the product on QIAxcel. As a result, we hypothesize that the specimens collected in AB might be *C. albertensis*, as this is the only species remaining in the *C. variipennis* complex with a geographical distribution in North America.

**Figure 2:**
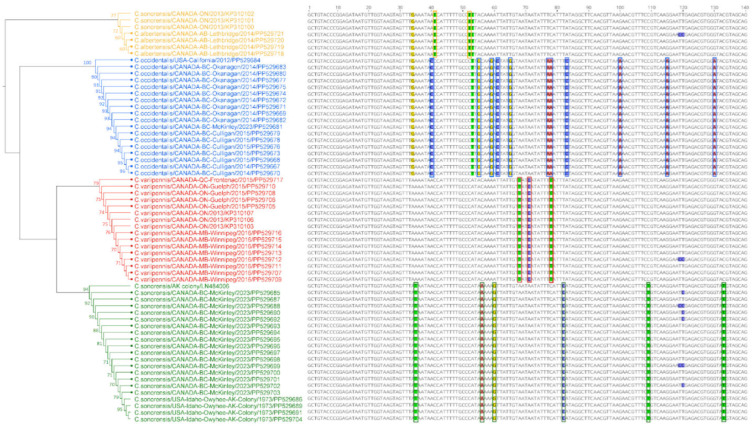
Phylogenetic tree and multiple-sequence alignment analysis for species of the *C. variipennis* complex using EF1α sequence data. Sequences derived from specimens collected for this study are shown with the addition of squares to branch tips. The topology shows Bayesian inference trees using Geneious Prime 2021.2.2. Only pairwise identity values above 70% are shown. Different species are identified by different colors.

The sequence alignment results of our QMono PCR products combined with the phylogeny tree revealed a unique collection of differentiating nucleotide patterns that serve as a species-specific nucleotide signature. Based on this nucleotide signature, sequences of *C. sonorensis* are unique in nucleotide positions 35 (T), 56 (A), 60 (G), 82 (C), 109 (T), and 133 (T), sequences of *C. variipennis* are unique in nucleotide positions 68 (T), 71 (C), and 78 (T), sequences of *C. occidentalis* are unique in nucleotide positions 40 (C), 55 (G), 59 (G), 61 (C), 65 (G), 77 and 78 (A), 83 (C), 100 (A), 115 (A), and 130 (A), and sequences of *C. albertensis* (putative) are unique in nucleotide positions 41 and 52 (T) (Figure 2).

### 3.6. Geographical Distribution of Species within the C. variipennis Complex in Canada

**Figure 3:**
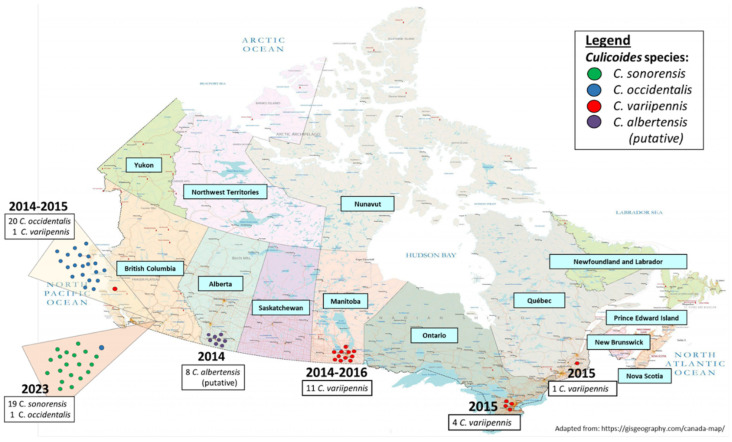
Distribution of a subset of *Culicoides* species within the *C. variipennis* complex across Canada from 2014–2016, including the direct comparison of changes in species between 2014–2015 and 2023 in British Columbia.

Our preliminary geographical dispersal findings using 65 field-collected specimens (60 specimens presented in Table 5 plus 4 *C. variipennis* specimens from ON and 1 *C. variipennis* from QC presented in Table 1 and Figure 1, respectively) using multiplex real-time PCR indicated that, out of the 21 archived specimens from BC, 20 specimens (95.2%) (10 from 2014 and 10 from 2015) were confirmed as *C. occidentalis*, and 1 specimen (4.7%) collected in 2014 was identified as *C. variipennis*. It was determined that all 11 specimens (100%) from MB—10 from 2016 and 1 from 2014—were confirmed as *C. variipennis*, and none of the 8 AB specimens collected in 2014 were determined as belonging to any of the three species (*C. sonorensis*, *C. occidentalis*, or *C. variipennis*) (Table 5). From Eastern Canada, four archived specimens from ON collected in 2015 (Table 1) and one archived specimen from QC collected in 2015 (Figure 2) were identified as *C. variipennis*. Furthermore, out of the 20 frozen specimens collected in BC in 2023, 19 specimens (95%) specimens were confirmed to be *C. sonorensis*, and 1 specimen (5%) was identified as *C. occidentalis* (Table 5). The geographical distribution of the 65 randomly selected specimens is shown in Figure 3.

## 4. Discussion

*Culicoides* biting midges are vectors of different veterinary viruses, such as BTV and EHDV, which cause significant economic losses [1,2]. Among the different species of *Culicoides*, *C. sonorensis* is one of the species of interest as it is considered the primary vector of BTV serotypes 10, 11, 13, and 17 in North America [28] and is also a confirmed EHDV vector [29]. The endemic area for BTV and EHDV in North America remains fluid, as *Culicoides* species have been shown to disperse via prevailing winds for hundreds of kilometers, crossing the border from the USA to Canada [11]. This emphasizes the significance and necessity of continuing surveillance programs for both the virus and its vectors, as Canada declares itself seasonally free of BTV and EHDV.

The most common method for the identification of *Culicoides* biting midge species is morphological identification; however, interpretation is challenging without specialized taxonomic expertise and is made even more difficult if distinguishing features of the specimen are damaged. Furthermore, mounting procedures for morphological identification can compromise RNA integrity, hindering subsequent arbovirus screening [30] and creating bottlenecks in large-scale surveillance projects due to the time-consuming nature of the process. While valuable for educational purposes, morphological identification is costly and labor-intensive, particularly for complex groups like *C. variipennis*. In contrast, molecular methods, although involving higher initial costs, offer significant advantages. They are less labor-intensive, require less expert training, and provide faster, more accurate, and scalable results. Given these benefits, molecular identification is recommended for efficient and precise classification of the *C. variipennis* complex, particularly in large-scale or high-precision contexts.

Due to the lack of comprehensive and reliable *Culicoides* surveillance efforts in Canada, the national distribution of *Culicoides* species serving as vectors for BTV and EHDV is poorly understood. As such, the distribution and composition of different species within the *C. variipennis* complex is incomplete, especially for *C. sonorensis*, the main vector for BTV and EHDV in North America [28,29]. Surveillance of *C. sonorensis* and its presence in various regions of Canada has been reported, for example, in ON [7] and AB [5], but data across other provinces is lacking. Therefore, the accurate identification of *C. sonorensis* in vector surveillance programs is essential for developing an efficient BTV and EHDV control policy. In this study, we established a novel multiplex real-time PCR method that is rapid, sensitive, and specific in the identification of polymorphic differences within the EF1α gene among three cryptic species: *C. sonorensis*, *C. variipennis*, and *C. occidentalis.* This tool is expected to be used for the species identification of large sample sizes in the field and surveillance programs for BTV and EHDV.

The use of COI barcoding is considered an effective tool to rapidly identify insects without the need for morphological identification, which requires immense expertise [31]. However, a previous study published by Shults et al. in 2022 found that mitochondrial genes, such as COI and others typically used for species identification through barcoding, are unable to discriminate between the species within the *C. variipennis* complex [11]. Similar results were also obtained by Hopken et al. in 2016 (33) using four other genes: nuclear carbamoyl phosphate synthetase, triose phosphate isomerase, and mitochondrial COI and COII genes. Their primary finding was that none of these four genes provided evidence for *C. sonorensis* and *C. variipennis* to be two distinct monophyletic groups [32]. Using other sequencing data, however, Shults et al. (2022) concluded that single nucleotide polymorphisms (SNPs) do exist, which can differentiate between *C. variipennis*, *C. occidentalis*, and *C. sonorensis*, molecularly. Furthermore, they identified the presence of two additional species in the complex: *C. albertensis* and a second cryptic species present in San Diego, California. Shults et al. (2022) expanded on their original study by developing a method using microsatellite markers to differentiate between the five species in the complex [33]. They tested 21 different markers, and their results demonstrated that 7 markers were sufficient to distinguish between all the species in the complex. Using microsatellites instead of sequencing is both less time-consuming and costly, but there is still the need for several PCRs and involved data analysis.

To address these challenges, we designed an assay to resolve the species in this complex using the same principle, the detection of SNPs, but with a different approach. Utilizing in-house designed QMono primers to amplify and sequence a short region of the EF1α gene, we created a phylogenetic tree showing the delimitation of three species (*C. sonorensis*, *C. occidentalis*, and *C. variipennis*) within the *C. variipennis* complex. The genetic differentiation between three cryptic species in this EF1α region proved the feasibility of a molecular tool to disambiguate cryptic species within the *C. variipennis* complex based on SNP detection within the EF1α gene. Our method, while based on the same principles, is unique compared to both previous approaches (sequencing and PCR) in that it can easily distinguish between all three species in one real-time PCR reaction, providing quick and reliable results without the need for post-PCR analysis (i.e., electrophoresis or sequencing). For vector surveillance programs, the method is straightforward and allows for the pooling of samples, which saves both time and money; only pools containing positive samples within the pool would require additional analysis to identify the positive sample inside the pool.

One tenet of any newly developed qPCR methodology is specificity; it is one of, if not the most crucial, requirements for the development of a methodology utilized for surveillance programs. Here, we describe the development of a new multiplex real-time PCR method that has a high specificity for the targeted species within the *C. variipennis* complex. The method was tested against various *Culicoides* species that have been identified in Canada, and the probes showed no cross-reactivity between any of the species tested. One factor that enhances the specificity of our qPCR is that both the probes and the primers were found to be largely specific for the *Culicoides* species within the *C. variipennis* complex. Like the findings of Shults et al. (2022) [33], the developed method targets SNPs to distinguish between the species in the complex and from other *Culicoides* species, resulting in a high degree of specificity that is clearly demonstrated by the lack of cross-reactivity across various species, including other members of the *C. variipennis* complex.

The second crucial requirement in a molecular tool for surveillance is sensitivity. Although more expensive than conventional PCR methodologies, real-time PCR is a more rapid and more sensitive molecular tool, providing advantages in monitoring programs [34]. Pooling samples has been suggested to reduce this cost but keep the advantages in sensitivity and specificity combined with rapid PCR analysis. One of the main challenges of working with midges compared to other insects is the relatively small size of midges; typically, they are less than 3 mm in length [22], which may result in a loss of signal in pooled samples. We tested the sensitivity of the assay by creating various pools (10×, 20×, and 30×) with our targeted species, and we were able to identify the *C. variipennis* species target correctly in all pools, demonstrating the sensitivity of the designed qPCR. We then expanded the sensitivity testing by overwhelming the system with only DNA from the three targets present in the pools (e.g., one *C. variipennis* and nine *C. sonorensis*), further demonstrating the sensitivity and specificity of the qPCR as each target was again correctly identified in the pools. These results, along with the specificity results, demonstrate the high specificity and sensitivity of the designed PCR, providing a valuable and efficient tool to identify species in the *C. variipennis* complex.

The method was subsequently validated against 60 field-collected samples morphologically identified as belonging to the complex. All 60 specimens were successfully identified as either *C. sonorensis*, *C. occidentalis*, or *C. variipennis* using species-specific probes, except for 8 AB 2014 samples, which were initially identified morphologically as belonging to the *C. variipennis* complex (Table 5). We believe this is confirmed by the fact that the AB specimens produced a QMono PCR product (149 bp in size), which, with the species we have tested, demonstrates that several *Culicoides* species outside the *C. variipennis* complex are incapable of generating a PCR product with our QMono primers (Table 2). As a result, our initial hypothesis was that the specimens found in AB could possibly be *C. albertensis* because, after excluding our probe-targeted species within the *C. variipennis* complex, *C. albertensis* is the only remaining species with an ecological niche in North America.

To test this theory, we performed a sequence analysis combining sequence alignments and a phylogenetic tree. Interestingly, data revealed that sequences derived from the AB specimens formed an outgroup distinct from the three other species as *C. variipennis*, *C. sonorensis*, and *C. occidentalis*. The accuracy of the phylogenetic tree was validated by including known *C. sonorensis*-only *Culicoides* colony sequences and reference sequences from GenBank. Moreover, the AB specimen-derived sequences displayed a specific-species nucleotide signature that set them apart from the other three species within the *C. variipennis* complex. Combining this finding with the geographical distribution of *C. albertensis* in North America [11], we postulate that unique sequences from AB should be *C. albertensis*. The morphological identification of the speculated *C. albertensis* in this study was not successful. This is because morphological discrimination between *C. sonorensis* and *C. albertensis* is very difficult and is based on the scutum of *Culicoides*, where spots are more clustered together in *C. sonorensis* and more spaced evenly spaced in *C. albertensis*. However, these spots have faded for the samples used in this study.

Since the probes are specifically designed only for *C. sonorensis*, *C. variipennis*, and *C. occidentalis*, it was expected that the suspected *C. albertensis* would not generate any signal in the multiplex real-time PCR. When analyzing the sequence of probe binding sites, there is a two-bp difference between *C. variipennis* and *C. albertensis*; a (T) to (C) difference at the 52nd position and an (A) to (T) difference at the 53rd position. For the *C. sonorensis* probe binding site, a four-bp difference is observed: two of these differences are the same SNPs observed in *C. variipennis*, an (A) to (C) difference at the 56th position, a (G) to (A) difference at the 60th position. For *C. occidentalis*, an (A) to (G) difference at the 115th position and a (G) to (A) difference at the 166th position are observed (Supplementary Figure S1).

The evolutionary tree analysis, unsurprisingly, revealed that the *C. variipennis* sequences (KP310103, KP310106, and KP310107) reported by A. Jewiss-Gaines et al. from ON in 2013 [7] clustered with other *C. variipennis* sequences derived from our specimens. What was interesting, though, was the grouping of three sequences collected from ON in 2013, previously reported as *C. sonorensis* (KP310100–KP310102) by A. Jewiss-Gaines et al. with the *C. albertensis* (putative) specimens collected in AB in 2014. The comparison of these previously reported *C. sonorensis* sequences with sequences derived from our field-collected *C. sonorensis* and known *C. sonorensis*-only *Culicoides* colony based on 140 bp shown in Figure 3 showed no similarity to nucleotide signatures specific to *C. sonorensis*. In contrast, three ON-2013 sequences from A. Jewiss-Gaines et al. studies were identical to our *C. albertensis* (putative) sequences at positions 41 and 52. In addition to the sequence analysis based on 140 bp shown in Figure 3, the comparison of *C. sonorensis* sequences reported by A. Jewiss-Gaines et al. based on longer length sequences with the reference sequence of *C. sonorensis* (LN484006) confirmed that sequences previously reported as *C. sonorensis* are not *C. sonorensis*, but actually *C. albertensis*. This claim is in accordance with previous data analyses by Shults et al. [11,33]. Although the current study did not aim at a systematic analysis of species within the *C. variipennis* complex habitats, based on SNP analysis of previously reported *C. sonorensis* in ON, and knowing that *C. albertensis* is designated as a synonym of *C. sonorensis* [11], we suggest the need for further detailed studies to update the previous occurrence reports of *C. sonorensis* in ON and AB, based on updated *C. variipennis* complex taxonomy [10].

The 65 specimens of the *C. variipennis* complex (Figure 3), initially identified by a morphological key, collected from BC, AB, MB, ON, and QC between 2014 and 2023 as part of a nationwide *Culicoides* biting midges surveillance program were subjected to the recently developed multiplex real-time PCR in order to discriminate the species within the *C. variipennis* complex. Although the main objective of the current study was to establish and validate a molecular method for the identification of species within the *C. variipennis* complex, and not the prevalence of species in different habitats, the experiment on a subset of *C. variipennis* complex specimens in Canada over different years showed a shift from *C. occidentalis* in 2014/2015 (95.2%) to *C. sonorensis* (95%) in 2023 in BC. Moreover, all specimens from AB and MB were identified as *C. albertensis* (putative) and *C. variipennis*, respectively. In addition, all specimens from two Eastern provinces of Canada, QC and ON, during our study were identified as *C. variipennis* (Figure 3).

The preliminary habitat dispersal findings indicate a dissimilar distribution of the *C. variipennis* complex throughout the country. This distribution includes the occurrence of *C. variipennis* mostly in Eastern-Central Canada, and *C. sonorensis* and *C. occidentalis* in Western Canada (Figure 3), which is in accordance with similar data from the USA, indicating *C. variipennis* in the Eastern USA, *C. sonorensis* in the Western and Southern USA, *C. occidentalis* in the North Western USA, and *C. albertensis* in the Midwest USA [11,12]. However, our findings and the spatial distribution of the *C. variipennis* complex in the USA by Shults et al. [11] are in contrast with previous findings in Canada, reporting the presence of Western *C. sonorensis* in ON in 2013 [7], and AB in 2002–2012 [5]. Since previous *C. sonorensis* reports from ON and AB were based on morphological features or standard barcoding procedures, which render the accuracy of species-level identification of synonymous species of *C. sonorensis* unreliable, the current study suggests revisiting previous *C. sonorensis* reports from Canada.

We know that the artificial range expansion of *C. sonorensis* in previous reports from Canada was probably due to the inability to distinguish three previously recognized species plus *C. albertensis* (previously synonymized with *C. sonorensis*) based on morphological features. Therefore, the molecular identification tool developed in the current study will be an asset for the accurate identification of cryptic species within the *C. variipennis* complex in future surveillance programs in North America. In addition, the results of the current study open new possibilities for investigation. Therefore, we hope to expand this assay to include *C. albertensis* and map the updated spatiotemporal distribution of *C. sonorensis* in Canada in our future research. We will achieve this by leveraging the large sample size of the *C. variipennis* complex at CFIA and the currently established multiplex real-time PCR.

## Figures and Tables

**Table 1:** Validation of multiplex real-time PCR for the C. *variipennis* complex reference specimens.

			Probe Fluorophore			
Samples	Collection Year	Collection Site	CY5— *C. sonorensis*	HEX— *C. variipennis*	FAM— *C. occidentalis*	TAMRA— 18S Internal Control
*C. sonorensis* #1	1973	Idaho, United States	+	−	−	+
*C. sonorensis* #2	1973	Idaho, United States	+	−	−	+
*C. sonorensis* #3	1973	Idaho, United States	+	−	−	+
*C. sonorensis* #4	1973	Idaho, United States	+	−	−	+
*C. variipennis* #1	2015	Ontario, Canada	−	+	−	+
*C. variipennis* #2	2015	Ontario, Canada	−	+	−	+
*C. variipennis* #3	2015	Ontario, Canada	−	+	−	+
*C. variipennis* #4	2015	Ontario, Canada	−	+	−	+
*C. occidentalis* #1	2015	British Columbia, Canada	−	−	+	+
*C. occidentalis* #2	2015	British Columbia, Canada	−	−	+	+
*C. occidentalis* #3	2015	British Columbia, Canada	−	−	+	+

Note: (+) symbol denotes the presence of a CT value for a sample, and (−) symbol denotes the absence of a CT value for a sample. Each fluorophore used in the multiplex real-time PCR is identified by a distinct color: CY5 (red), HEX (green), FAM (blue), and TAMRA (white).

**Table 2:** Specificity of multiplex real-time PCR by comparing *C. variipennis* complex species versus non-*C. variipennis* complex species collected in Canada.

Samples	Collection Year	Collection Site	Probe Fluorophore			
			CY5— *C. sonorensis*	HEX— *C. variipennis*	FAM— *C. occidentalis*	TAMRA—18S Internal Control
subgenus *Avaritia* *	2016	Nova Scotia, Canada	−	−	−	+
subgenus *Avaritia* **	2016	Nova Scotia, Canada	−	−	−	+
*C. biguttatus*	2016	Nova Scotia, Canada	−	−	−	+
*C. travisi*	2022	British Columbia, Canada	−	−	−	+
*C. stellifer*	2022	Ontario, Canada	−	−	−	+
*C. crepuscularis*	2014	British Columbia, Canada	−	−	−	+
*C. yukonensis*	2014	Saskatchewan, Canada	−	−	−	+
*C. downesi*	2015	Nova Scotia, Canada	−	−	−	+
*C. riethi*	2017	Alberta, Canada	−	−	−	+
*C. wisconsinensis*	2014	Alberta, Canada	−	−	−	+
*C. denticulatus*	2015	Nova Scotia, Canada	−	−	−	+
*C. paraimpunctatus*	2016	Nova Scotia, Canada	−	−	−	+
*C. haematopotus*	2015	Ontario, Canada	−	−	−	+
*C. cockerellii*	2022	Saskatchewan, Canada	−	−	−	+
*piliferus* species group ***	2022	Quebec, Canada	−	−	−	+
*C. occidentalis*	2015	British Columbia, Canada	−	−	+	+
*C. sonorensis*	2015	Idaho, United States	+	−	−	+
*C. variipennis*	2015	Ontario, Canada	−	+	−	+

Note: * *C. sanguisuga* based on COI barcoding (GenBank), ** *C. obsoletus* based on COI barcoding (GenBank), *** *C. scanloni* based on COI barcoding (BOLD), (+) symbol denotes the presence of a CT value for a sample, (−) symbol denotes the absence of a CT value for a sample. Each fluorophore used in the multiplex real-time PCR is identified by a distinct color: CY5 (red), HEX (green), FAM (blue), and TAMRA (white).

**Table 3:** Multiplex qPCR of *C. variipennis* complex specimens spiked into non-*C. variipennis* complex sample pools.

		Probe Fluorophore			
	Sample Pools	CY5—*C. sonorensis*	HEX—*C. variipennis*	FAM—*C. occidentalis*	TAMRA—18S Internal Control
Sensitivity	9 *Culicoides* plus 1 *C. occidentalis*	−	−	34.95	27.58
	19 *Culicoides* plus 1 *C. occidentalis*	−	−	36.23	27.66
	29 *Culicoides* plus 1 *C. occidentalis*	−	−	36.07	28.14
	9 *Culicoides* plus 1 *C. sonorensis*	28.63	−	−	28.53
	19 *Culicoides* plus 1 *C. sonorensis*	29.3	−	−	29.02
	29 *Culicoides* plus 1 *C. sonorensis*	29.89	−	−	28.46
	9 *Culicoides* plus 1 *C. variipennis*	−	28.97	−	27.41
	19 *Culicoides* plus 1 *C. variipennis*	−	29.96	−	27.98
	29 *Culicoides* plus 1 *C. variipennis*	−	30.31	−	28.65
	*C. occidentalis* 1/10 dilution	−	−	35.46	33.38
	*C. occidentalis* 1/20 dilution	−	−	36.23	34.69
	*C. occidentalis* 1/30 dilution	−	−	37.13	35.15
	*C. sonorensis* 1/10 dilution	28.56	−	−	28.31
	*C. sonorensis* 1/20 dilution	29.66	−	−	29.22
	*C. sonorensis* 1/30 dilution	29.93	−	−	29.81
	*C. variipennis* 1/10 dilution	−	28.8	−	24.24
	*C. variipennis* 1/20 dilution	−	29.88	−	27.87
	*C. variipennis* 1/30 dilution	−	30.42	−	28.65
	9 *Culicoides* plus water	−	−	−	27.23
	19 *Culicoides* plus water	−	−	−	27.78
	29 *Culicoides* plus water	−	−	−	28.35

Note: (−) symbol denotes the absence of a CT value for a sample. *Culicoides* refers to any *Culicoides* species that is not part of the *C. variipennis* complex (*C. sonorensis*, *C. variipennis* and *C. occidentalis*). Each fluorophore used in the multiplex real-time PCR is identified by a distinct color: CY5 (red), HEX (green), FAM (blue), and TAMRA (white).

**Table 4:** Multiplex real-time PCR of pooled *C. variipennis* complex species with spiked-in *C. variipennis*-complex targets.

		Probe Fluorophore			
	Sample Pools	CY5—*C. sonorensis*	HEX—*C. variipennis*	FAM—*C. occidentalis*	TAMRA— 18S Internal Control
Sensitivity	9 *C. sonorensis* plus 1 *C. occidentalis*	25.4	−	29.86	23.21
	*8 C. sonorensis* plus 2 *C. occidentalis*	25.46	−	29.2	23.15
	*7 C. sonorensis* plus 3 *C. occidentalis*	25.7	−	29.2	23.43
	9 *C. variipennis* plus 1 *C. occidentalis*	−	26.85	29.21	26.22
	8 *C. variipennis* plus 1 *C. occidentalis*	−	26.64	28.83	26.44
	7 *C. variipennis* plus 3 *C. occidentalis*	−	26.95	28.87	26.7
	9 *C. sonorensis* plus 1 *C. variipennis*	25.06	34.53	−	23.15
	8 *C. sonorensis* plus 2 *C. variipennis*	24.95	28.17	−	22.79
	7 *C. sonorensis* plus 3 *C. variipennis*	25.49	28.06	−	23.22
	9 *C. occidentalis* plus 1 *C. variipennis*	−	29.32	28.53	27.55
	8 *C. occidentalis* plus 2 *C. variipennis*	−	26.14	29.11	24.97
	7 *C. occidentalis* plus 3 *C. variipennis*	−	27.72	28.67	26.42
	9 *C. occidentalis* plus 1 *C. sonorensis*	29.64	−	28.57	28.97
	8 *C. occidentalis* plus 2 *C. sonorensis*	28.22	−	28.71	27.98
	7 *C. occidentalis* plus 3 *C. sonorensis*	27.63	−	28.59	27.32
	9 *C. variipennis* plus 1 *C. sonorensis*	29.9	25.82	−	23.06
	8 *C. variipennis* plus 2 *C. sonorensis*	27.82	25.33	−	22.43
	7 *C. variipennis* plus 3 *C. sonorensis*	26.87	25.16	−	22.12
	1 *C. sonorensis*, 1 *C. variipennis* and 1 *C. occidentalis*	30.35	28.2	29.56	25.96
	2 *C. sonorensis*, 2 *C. variipennis* and 2 *C. occidentalis*	28.41	27.96	29.34	25.87
	3 *C. sonorensis*, 3 *C. variipennis* and 3 *C. occidentalis*	27.87	27.4	29.27	25.34
	3 *C. occidentalis* plus 7× water	−	−	28.97	28.02
	2 *C. occidentalis* plus 8× water	−	−	29.22	27.95
	1 *C. occidentalis* plus 9× water	−	−	29.23	27.65
	3 *C. sonorensis* plus 7× water	26.34	−	−	24.46
	2 *C. sonorensis* plus 8× water	27.46	−	−	26.32
	1 *C. sonorensis* plus 9× water	28.75	−	−	26.47
	3 *C. variipennis* plus 7× water	−	26.93	−	23.99
	2 *C. variipennis* plus 8× water	−	27.53	−	24.6
	1 *C. variipennis* plus 9× water	−	29.34	−	25.21

Note: (−) symbol denotes the absence of a CT value for a sample. Each fluorophore used in the multiplex real-time PCR is identified by a distinct color: CY5 (red), HEX (green), FAM (blue), and TAMRA (white).

**Table 5:** Species identification of field-collected *C. variipennis*-complex species in Canada (2014–2016, 2023).

				Probe Fluorophore			
	Samples	Collection Year	Collection Site	CY5— *C. sonorensis*	HEX— *C. variipennis*	FAM— *C. occidentalis*	TAMRA— 18S Internal Control
Archived Samples (Ethanol Preserved)	MB #1	2014	Manitoba, Canada	−	+	−	+
	MB #2	2016	Manitoba, Canada	−	+	−	+
	MB #3	2016	Manitoba, Canada	−	+	−	+
	MB #4	2016	Manitoba, Canada	−	+	−	+
	MB #5	2016	Manitoba, Canada	−	+	−	+
	MB #6	2016	Manitoba, Canada	−	+	−	+
	MB #7	2016	Manitoba, Canada	−	+	−	+
	MB #8	2016	Manitoba, Canada	−	+	−	+
	MB #9	2016	Manitoba, Canada	−	+	−	+
	MB #10	2016	Manitoba, Canada	−	+	−	+
	MB #11	2016	Manitoba, Canada	−	+	−	+
	AB #1	2014	Alberta, Canada	−	−	−	+
	AB #2	2014	Alberta, Canada	−	−	−	+
	AB #3	2014	Alberta, Canada	−	−	−	+
	AB #4	2014	Alberta, Canada	−	−	−	+
	AB #5	2014	Alberta, Canada	−	−	−	+
	AB #6	2014	Alberta, Canada	−	−	−	+
	AB #7	2014	Alberta, Canada	−	−	−	+
	AB #8	2014	Alberta, Canada	−	−	−	+
	BC #1	2014	British Columbia, Canada	−	−	+	+
	BC #2	2014	British Columbia, Canada	−	−	+	+
	BC #3	2014	British Columbia, Canada	−	−	+	+
	BC #4	2014	British Columbia, Canada	−	−	+	+
	BC #5	2014	British Columbia, Canada	−	−	+	+
	BC #6	2014	British Columbia, Canada	−	−	+	+
	BC #7	2014	British Columbia, Canada	−	−	+	+
	BC #8	2014	British Columbia, Canada	−	−	+	+
	BC #9	2014	British Columbia, Canada	−	−	+	+
	BC #10	2014	British Columbia, Canada	−	−	+	+
	BC #11	2015	British Columbia, Canada	−	−	+	+
	BC #12	2015	British Columbia, Canada	−	−	+	+
	BC #13	2015	British Columbia, Canada	−	−	+	+
	BC #14	2015	British Columbia, Canada	−	−	+	+
	BC #15	2015	British Columbia, Canada	−	−	+	+
	BC #16	2015	British Columbia, Canada	−	−	+	+
	BC #17	2015	British Columbia, Canada	−	−	+	+
	BC #18	2015	British Columbia, Canada	−	−	+	+
	BC #19	2015	British Columbia, Canada	−	−	+	+
	BC #20	2015	British Columbia, Canada	−	−	+	+
	BC #21	2014	British Columbia, Canada	−	+	−	+
Frozen Samples (−70 °C)	BC #22	2023	British Columbia, Canada	+	−	−	+
	BC #23	2023	British Columbia, Canada	+	−	−	+
	BC #24	2023	British Columbia, Canada	+	−	−	+
	BC #25	2023	British Columbia, Canada	+	−	−	+
	BC #26	2023	British Columbia, Canada	+	−	−	+
	BC #27	2023	British Columbia, Canada	+	−	−	+
	BC #28	2023	British Columbia, Canada	+	−	−	+
	BC #29	2023	British Columbia, Canada	+	−	−	+
	BC #30	2023	British Columbia, Canada	+	−	−	+
	BC #31	2023	British Columbia, Canada	+	−	−	+
	BC #32	2023	British Columbia, Canada	+	−	−	+
	BC #33	2023	British Columbia, Canada	−	−	+	+
	BC #34	2023	British Columbia, Canada	+	−	−	+
	BC #35	2023	British Columbia, Canada	+	−	−	+
	BC #36	2023	British Columbia, Canada	+	−	−	+
	BC #37	2023	British Columbia, Canada	+	−	−	+
	BC #38	2023	British Columbia, Canada	+	−	−	+
	BC #39	2023	British Columbia, Canada	+	−	−	+
	BC #40	2023	British Columbia, Canada	+	−	−	+
	BC #41	2023	British Columbia, Canada	+	−	−	+
Controls	*C. occidentalis*	2015	British Columbia, Canada	−	−	+	+
	*C. sonorensis*	2015	Idaho, United States	+	−	−	+
	*C. variipennis*	2015	Ontario, Canada	−	+	−	+

Note: (+) symbol denotes the presence of a Ct value for a sample, and the (−) symbol denotes the absence of a Ct value for a sample. Each fluorophore used in the multiplex real-time PCR is identified by a distinct color: CY5 (red), HEX (green), FAM (blue), and TAMRA (white).

## Data Availability

Data are contained within the article and Supplementary Materials.

## Supplementary Materials

The following supporting information can be downloaded at: https://www.mdpi.com/article/10.3390/cimb46090566/s1, Figure S1: Probe binding site of the species-specific probes. *C. sonorensis* and *C. variipennis* have the same binding site at positions 48–62 (red arrows), while *C. occidentalis* probe binding site is at positions 106–117 (blue arrows) within the 140 bp amplified region of the EF1α.
