# Supplementary material for: Development of a Multiplex Real-Time PCR to Disambiguate Culicoides sonorensis within Culicoides variipennis Complex, the Proven Vector of Bluetongue and Epizootic Hemorrhagic Disease Viruses in North America
Source: Curr Issues Mol Biol. 2024 Aug 29;46(9):9534–54. doi: 10.3390/cimb46090566 (PMC11429534; doi:10.3390/cimb46090566)
Supplement: Supplementary file 1 [file cimb-46-00566-s001.zip › cimb-3159470-supplementary.pdf]

Supplementary Materials

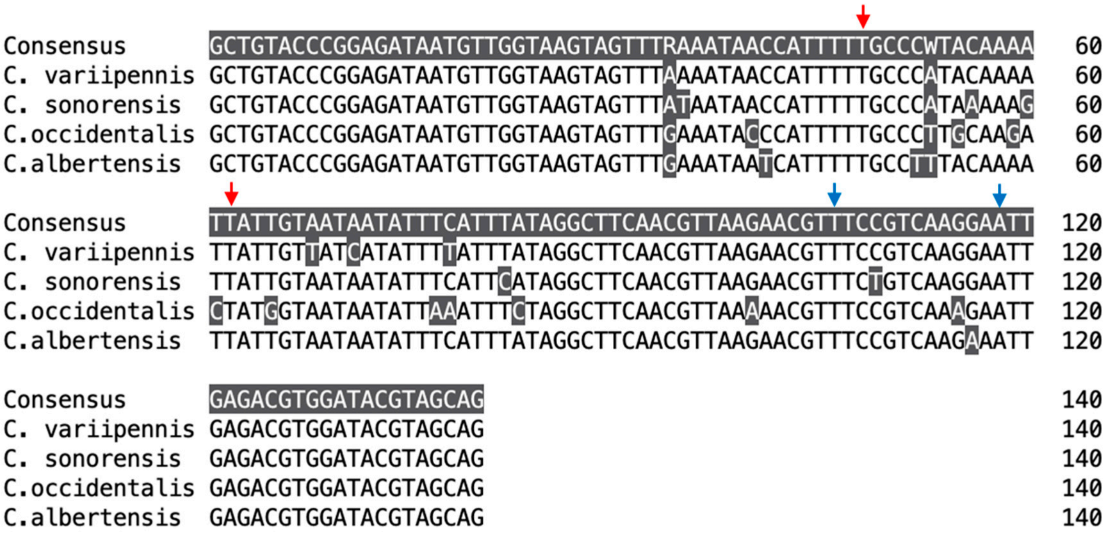

Figure S1. Probe binding site of the species-specific probes. *C. sonorensis* and *C. variipennis* have the same binding site at position 48–62 (red arrows) while *C. occidentalis* probe binding site is at position 106–117 (blue arrows) within the 140 bp amplified region of the EF1α.
